# Spondylodiscitis following endovascular abdominal aortic aneurysm repair: imaging perspectives from a single centre’s experience

**DOI:** 10.1007/s00256-018-2939-z

**Published:** 2018-04-14

**Authors:** Ramin Mandegaran, Christopher S. W. Tang, Erlick A. C. Pereira, Ali Zavareh

**Affiliations:** 1 Department of Radiology, Guy’s and St Thomas’ NHS Foundation Trust, 2nd Floor Tower Wing, Great Maze Pond, London, SE1 9RT UK; 2 Academic Neurosurgery Unit St George’s, University of London, London, SW17 0QT UK

**Keywords:** Spondylodiscitis, EVAR, MRI, PET/CT

## Abstract

**Objective:**

Very few reports have previously described spondylodiscitis as a potential complication of endovascular aortic aneurysm repair (EVAR). We present to our knowledge the first case series of spondylodiscitis following EVAR based on our institution’s experience over an 11-year period. Particular attention is paid to the key imaging features and challenges encountered when performing spinal imaging in this complex patient group.

**Materials and Methods:**

Of 1,847 patients who underwent EVAR at our institution between January 2006 and January 2017, a total of 9 patients were identified with imaging features of spondylodiscitis (0.5%). All cross-sectional studies before and after EVAR were assessed by a Consultant Musculoskeletal Radiologist and a Musculoskeletal Radiology Fellow to evaluate for features of spondylodiscitis.

**Results:**

All 9 patients had single-level spondylodiscitis involving lumbosacral levels adjacent to the aortic/iliac stent graft. Eight out of nine patients had an extensive anterior paravertebral phlegmon/abscess that was contiguous with the infected stent graft and native aneurysm sac ± anterior vertebral body erosion. Epidural disease was present in only 3 out of 9 patients and was a minor feature. MRI was non-diagnostic in 3 out of 9 patients owing to susceptibility artefact. ^18^F-FDG PET/CT accurately depicted the spinal level involved and adjacent paravertebral disease in patients with non-diagnostic MRI and was adopted as the follow-up modality in 3 out of 5 surviving patients.

**Conclusion:**

Spondylodiscitis is a rare complication post-EVAR. Imaging features of disproportionate anterior paravertebral disease and anterior vertebral body bony involvement suggest direct spread of infection posteriorly to the adjacent vertebral column. Use of MRI versus ^18^F-FDG PET/CT as the optimal imaging modality should be directed by the type of stent graft deployed.

## Introduction

Since it was first described in 1991 [1], endovascular aortic aneurysm repair (EVAR) has established itself over open surgical repair as the preferred method for treating abdominal aortic aneurysms in patients with suitable anatomy, given its lower mortality rate and comparable long-term survival [2, 3]. Whilst aortic graft infection as a complication of EVAR has been well documented in recent years, numerous studies have shown it to be a relatively rare problem, with an incidence of 0.5–1% [4–8].

Spondylodiscitis as a potential complication of EVAR has received very little attention in the literature and to our knowledge, only 4 case reports have been published to date [9–12]. We present a case series of 9 patients who went on to develop spondylodiscitis following EVAR, paying particular attention to: imaging findings and trends identified in the imaging features and discussing the challenges and optimal imaging strategy in this complex patient group, which is highly influenced by the type of stent graft deployed.

## Materials and methods

A retrospective analysis of all patients who underwent EVAR at a single tertiary centre over an 11-year period between January 2006 and January 2017 was performed to identify patients with features of spondylodiscitis on cross-sectional imaging studies after EVAR. A total of 1,442 abdominal EVARs and 405 thoracic EVARs (1,847 EVARs in total) were performed over the 11-year period. A total of 9 patients (0.5%) were identified with imaging features of spondylodiscitis on cross-sectional imaging (comprising CT, MRI and ^18^F-fluorodeoxyglucose [FDG] PET-CT studies) after EVAR. For each patient, all cross-sectional studies available, both before and after EVAR, were reviewed by a Consultant Musculoskeletal Radiologist and a Musculoskeletal Radiology Fellow to evaluate for the key features of spondylodiscitis (the presence of endplate erosion, disc enhancement, disc abscess) and the extent of paraspinal disease (the presence of an epidural or paravertebral phlegmon/abscess and associated psoas involvement). Features considered suggestive of a direct spread of infection to the spine following EVAR comprised involvement of spinal levels immediately adjacent to the stented aorto-iliac vessels; anterior paravertebral disease contiguous with the stent graft/native aneurysm sac; extensive erosion of the anterior vertebral body margin. Progression/resolution of imaging findings was also assessed on interval studies where available.

## Results

### Clinical features and diagnosis

All 9 patients were male, with a mean age of 73 years (range 58–87 years). The main clinical features for all 9 patients, are outlined in Table 1.

**Table 1:** Summary of clinical details, including initial aortic pathological condition, level of aortic stenting, initial presentation, time interval leading to identification of spondylodiscitis, and microbiological culture results

Patient	Initial aortic pathological condition	Location of stent	Presentation leading to identification of spondylodiscitis	Interval from stenting to first imaging features of spondylodiscitis (months)	Microbiological culture (source of culture sample)
1	Infra-renal AAA	Infra-renal aorta to bilateral CIAs	Central abdominal pain, vomiting	19	Culture-negative (spinal biopsy, blood culture)
2	Penetrating infra-renal ulcer	Infra-renal aorta stented initially—displaced inferiorly. Re-stented infra-renal aorta to bilateral CIAs	Back pain	6	*S. aureus* (adjacent psoas collection)
3	Infra-renal AAA	Infra-renal aorta to bilateral CIAs	Vomiting, fevers and rigours. Bony vertebral erosion noted at surgery during removal of infected stent graft.	2	*E. coli* (blood culture)
4	Infra-renal AAA	Infra-renal aorta to bilateral CIAs initially stented. Aortic stent collapsed—new infra-renal aortic stent deployed within pre-existing stent after 6 weeks	Lower back and right leg pain	3	Culture-negative (spinal biopsy, blood culture)
5	Infra-renal mycotic AAA (*E. coli* present in pre-EVAR blood culture)	Infra-renal aorta to bilateral CIAs	Asymptomatic. Vertebral endplate erosion on surveillance CT	2	*E. coli* (paravertebral collection)
6	Right CIA aneurysm	Infra-renal aorta to bilateral CIAs	Asymptomatic. Vertebral endplate erosion on surveillance CT	1	*S. pneumoniae* (blood culture)
7	Infra-renal AAA	Infra-renal aorta to bilateral CIAs	Fever	4	*S. aureus* (paravertebral collection)
8	Right IIA aneurysm	Infra-renal aorta to bilateral CIAs and right EIA (right IIA excluded by stent)	Lower back pain	6	*E. coli* (blood culture and adjacent psoas collection)
9	Suprarenal right-sided saccular AAA	Supra-renal abdominal aorta to bilateral CIAs with fenestrations for SMA and renal arteries	Fever	1	Culture-negative (paravertebral collection, blood culture)

*AAA* abdominal aortic aneurysm, *EVAR* endovascular aortic aneurysm repair, *CIA* common iliac artery, *IIA* internal iliac artery, *EIA* external iliac artery, *SMA* superior mesenteric artery

Eight patients had infra-renal EVAR for an infra-renal aortic/iliac artery pathological condition (7 patients with infra-renal aortic aneurysms, 1 patient with an infra-renal aortic penetrating ulcer and 1 patient with right internal iliac artery aneurysm). One patient with an infra-renal saccular aneurysm had a positive blood culture before EVAR, supporting the diagnosis of a probable infective aneurysm (patient 5). Another patient had a supra-renal aortic aneurysm requiring supra-renal extension of the EVAR (patient 9).

Clinical presentation following EVAR that led to imaging and identification of spondylodiscitis was variable. Back pain was the presenting feature in only 3 out of 9 patients. A further 3 out of 9 patients presented with non-specific features of sepsis (fever, rigours ± nausea and vomiting) and no significant pain. Of the remaining 3 patients, 1 complained of central abdominal pain and vomiting, whereas 2 patients were completely asymptomatic with vertebral endplate erosion noted incidentally on routine post-EVAR surveillance CT. Interval from initial aortic intervention to the first identification of spondylodiscitis features on imaging was also markedly variable, ranging from 1 to 19 months (median 3 months).

Positive microbiological cultures were identified in 6 out of 9 patients, all of which were bacterial (*Escherichia coli* in 3 patients, *Staphylococcus aureus* in 2 patients, *Streptococcus pneumoniae* in 1 patient). Two patients had positive cultures obtained from paravertebral collection biopsies and 2 patients from adjacent psoas abscess biopsy. Four patients had positive blood cultures (2 of whom additionally had positive cultures from paravertebral collection/psoas abscess biopsy). Spinal biopsy of the disc level involved was performed in 2 patients, both of whom were culture-negative. No causative organism was identified in 3 out of 9 patients.

### Imaging findings

The key imaging features of spondylodiscitis identified in all 9 patients are outlined in Table 2. In all 9 patients, only one single level lumbosacral disc involvement was demonstrated. L2/3 and L5/S1 were the most superior and inferior levels involved, with no involvement of spinal levels that were not immediately adjacent to the stent graft.

**Table 2:** Summary of spondylodiscitis imaging findings, progress and outcomes

Patient	Imaging modalities demonstrating spondylodiscitis features	Imaging features								Progress	Outcome
		Spondylodiscitis					Paraspinal phlegmon/abscess				
		Spinal level(s) involved	End plate erosion	Anterior vertebral body erosion	Disc enhancement/uptake	Disc abscess	Paravertebral	Psoas	Epidural		
1	CT, MRI	L2/3	+	+	+	+	+	+	+	Stent removal + axillobifemoral bypass. Progressive increase in paravertebral/discal abscess over 5 months	Died 7 months following identification of spondylodiscitis
2	PET/CT, MRI	L3/4	+	+	+	+	+	+	–	Left psoas collection aspirated—partial reduction in volume	Died 2 months following identification of spondylodiscitis (under palliative care for recurrent pharyngeal squamous cell carcinoma)
3	MRI, CT	L4/5	+	+	+	+	+	+	–	Stent removal + axillobifemoral bypass	Died 3 months following identification of spondylodiscitis
4	MRI	L5/S1	+	–	+	+	–	–	+	Resolution of disc abscess and epidural phlegmon on serial MRI	Discharged
5	CT, MRI, PET/CT	L4/5	+	+	+	–	+	–	+	Stent graft removal with complex aortic reconstruction. Paravertebral collection drained to resolution. L4/5 debridement, decompression and fusion—serial subsequent PET/CT shows no significant uptake at the fusion site	Discharged
6	CT, MRI, PET/CT	L4/5	+	–	+	–	+	–	–	Not followed up with imaging, cross sectional imaging	Discharged
7	CT, MRI	L2/3	+	+	+	+	+	+	–	Attempted drainage of the paravertebral collection	Died 1 month following identification of spondylodiscitis
8	CT, MRI	L5/S1	+	+	+	–	+	+	–	Attempted drainage of the right psoas collection—minimal volume aspirated	Discharged with follow-up PET/CT surveillance—did not attend
9	CT, MRI, PET/CT	L1/2	+	+	+	–	+	–	–	Reduction in the size of the paravertebral collection and uptake related to collection and disc space on interval PET/CT	Discharged with follow-up PET/CT surveillance

**Fig. 1:**
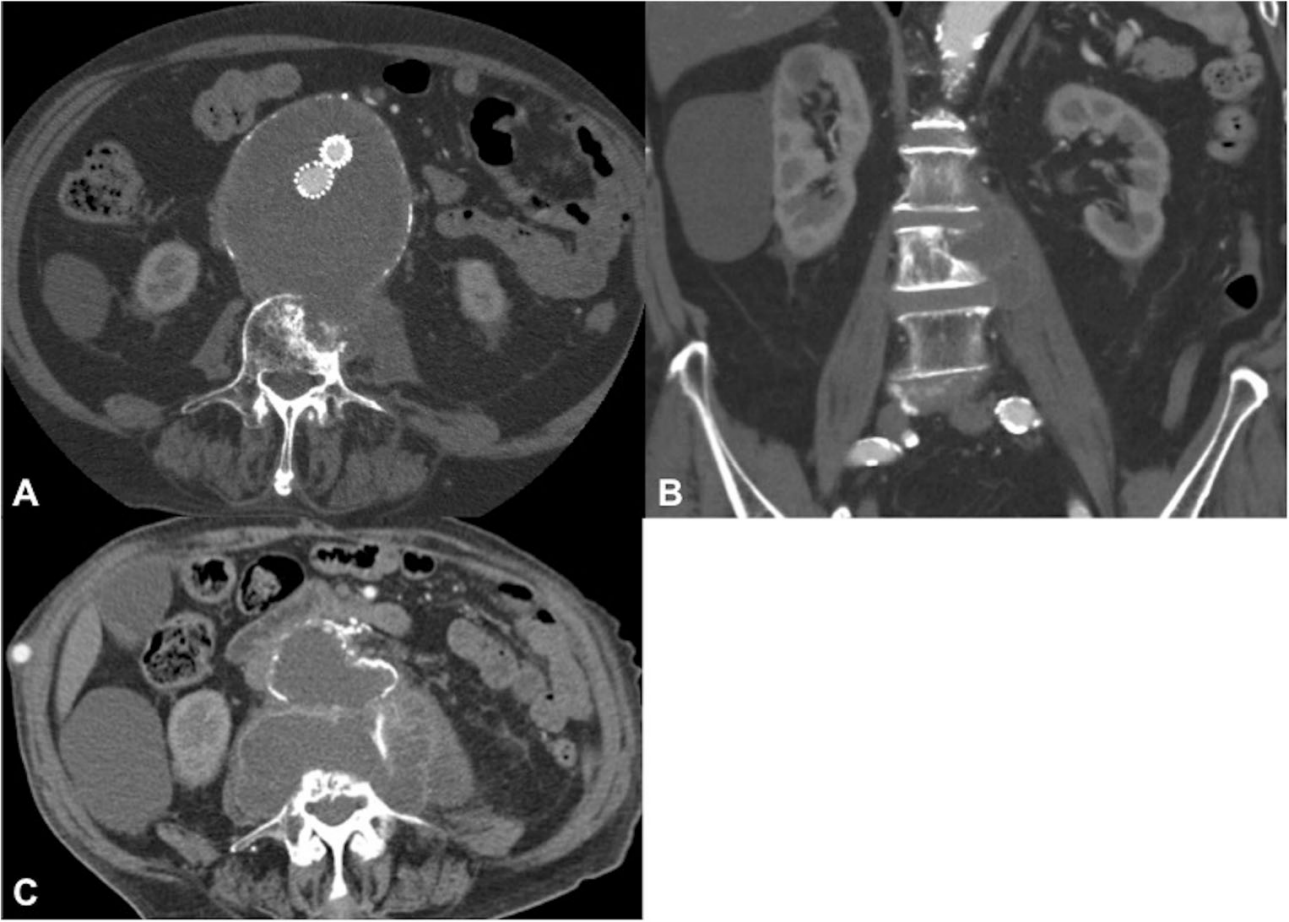
An 87-year-old man (patient 1). **a** Axial and **b** coronal CT 19 months following endovascular aortic aneurysm repair (EVAR), demonstrating L3 anterolateral vertebral body and superior endplate erosion. Peripherally enhancing paravertebral/left psoas abscess is contiguous with the markedly expanded native aneurysm encasing the stent graft. **c** Axial CT after a further 12 months with interval stent graft removal and axillobifemoral bypass. There is now more progressive vertebral body destruction with a contiguous abscess now involving the native excluded aneurysm sac, paravertebral soft tissues and replacing both psoas muscles

**Fig. 2:**
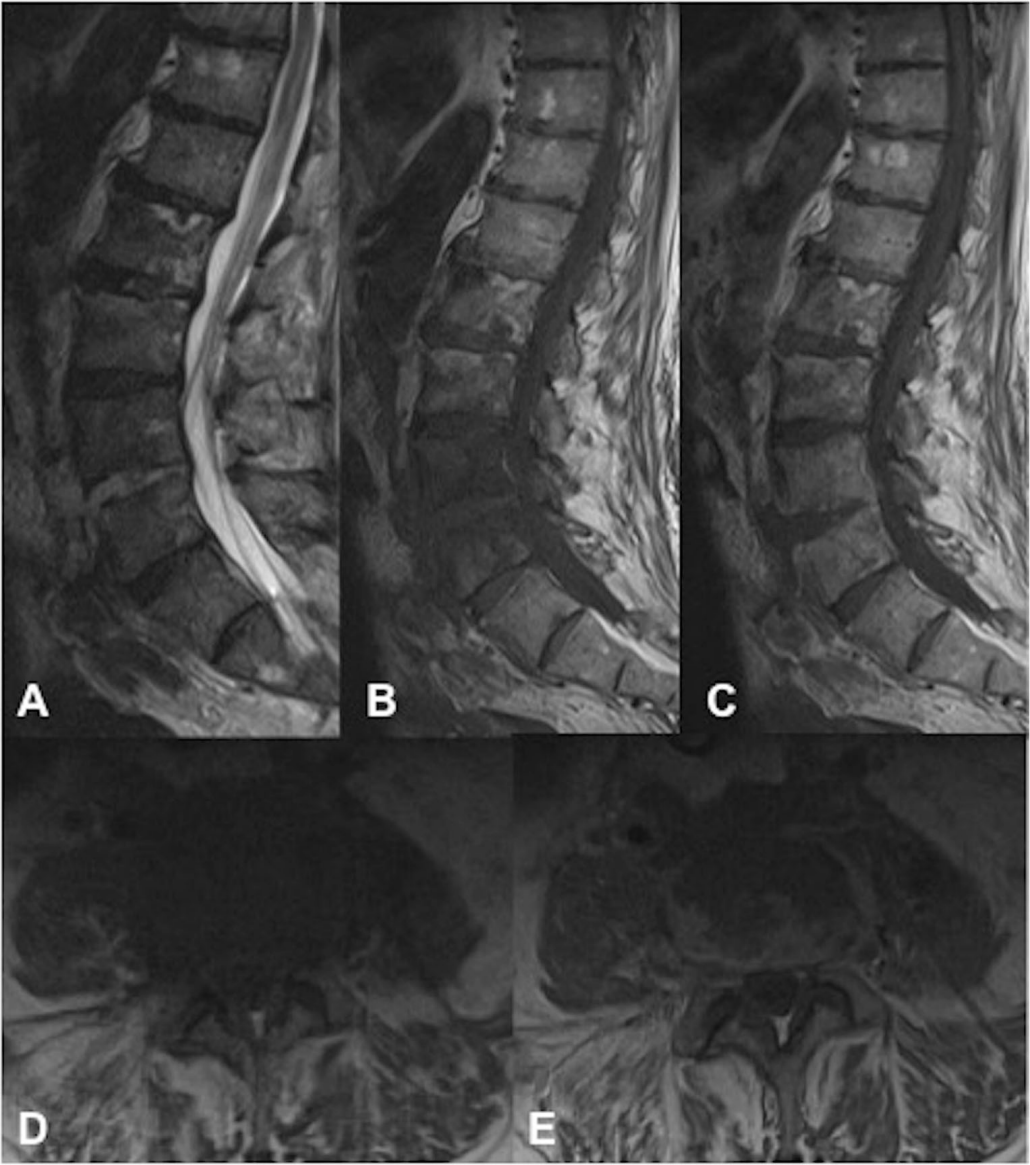
A 70-year-old man (patient 3). **a** Sagittal T2, **b** T1 pre-contrast and **c** T1 post-contrast sequences demonstrating increased T2-weighted signal centred on the L4/5 disc with anterior paravertebral extension corresponding to a peripherally enhancing abscess. **d** Axial T1 pre-contrast and **e** T1 post-intravenous contrast medium at the L4/5 disc level show contiguous peripherally enhancing disc/anterior paravertebral abscess. Note the lack of extension posterior to the epidural space

**Fig. 3:**
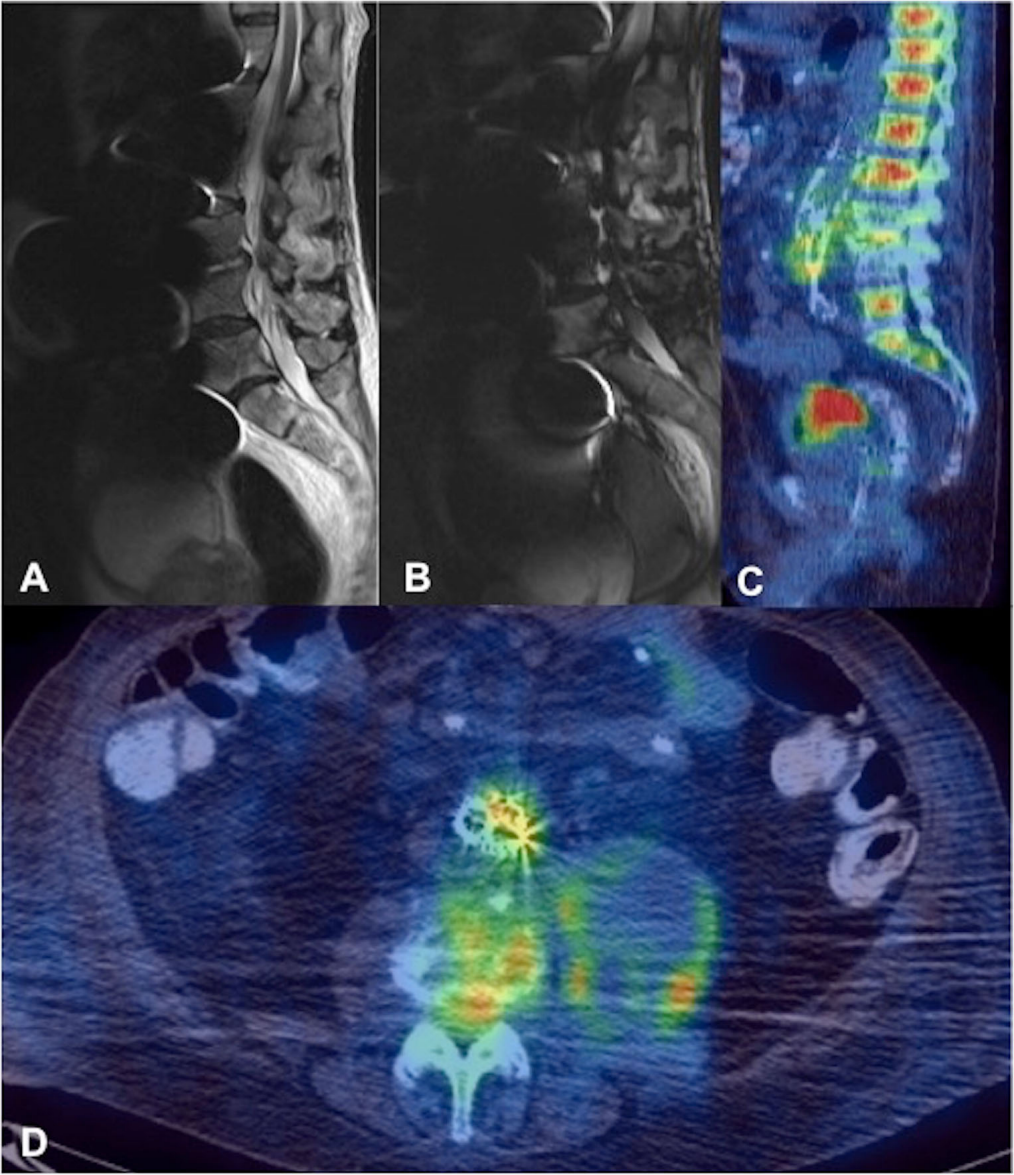
A 75-year-old man (patient 2). **a** Sagittal T2 and **b** STIR sequences demonstrating marked susceptibility artefact limiting image interpretation. Increased signal can be seen in the L3/4 disc in **a**, but no assessment of the paravertebral soft tissues can be made. **c** Sagittal and **d** axial PET/CT demonstrate abnormal increased uptake related to the EVAR stent graft with soft-tissue density and increased uptake extending into the L3/4 disc space, where there is endplate erosion and loss of disc height consistent with spondylodiscitis. Expansion of the left psoas with peripheral uptake suggests associated psoas abscess formation. Diffusely increased marrow uptake elsewhere in the spine was thought to be reactive

In 5 out of 9 patients, spondylodiscitis features were first identified on CT, demonstrating endplate erosion ± anterior vertebral body erosion, loss of disc height and paravertebral soft tissue/abscess (Fig. 1). All patients had at least one MRI spine (non-diagnostic in 2 patients and difficult to interpret in a third patient owing to extensive susceptibility artefact arising from the adjacent stent graft) and 4 patients underwent at least one ^18^F-FDG PET/CT (2 of whom had extensive artefact on MRI). All patients showed evidence of vertebral endplate erosion and either intervertebral disc enhancement on MRI (Fig. 2) or markedly increased tracer uptake on ^18^F-FDG PET/CT (Fig. 3), although only 5 out of 9 had imaging evidence of a disc abscess.

**Fig. 4:**
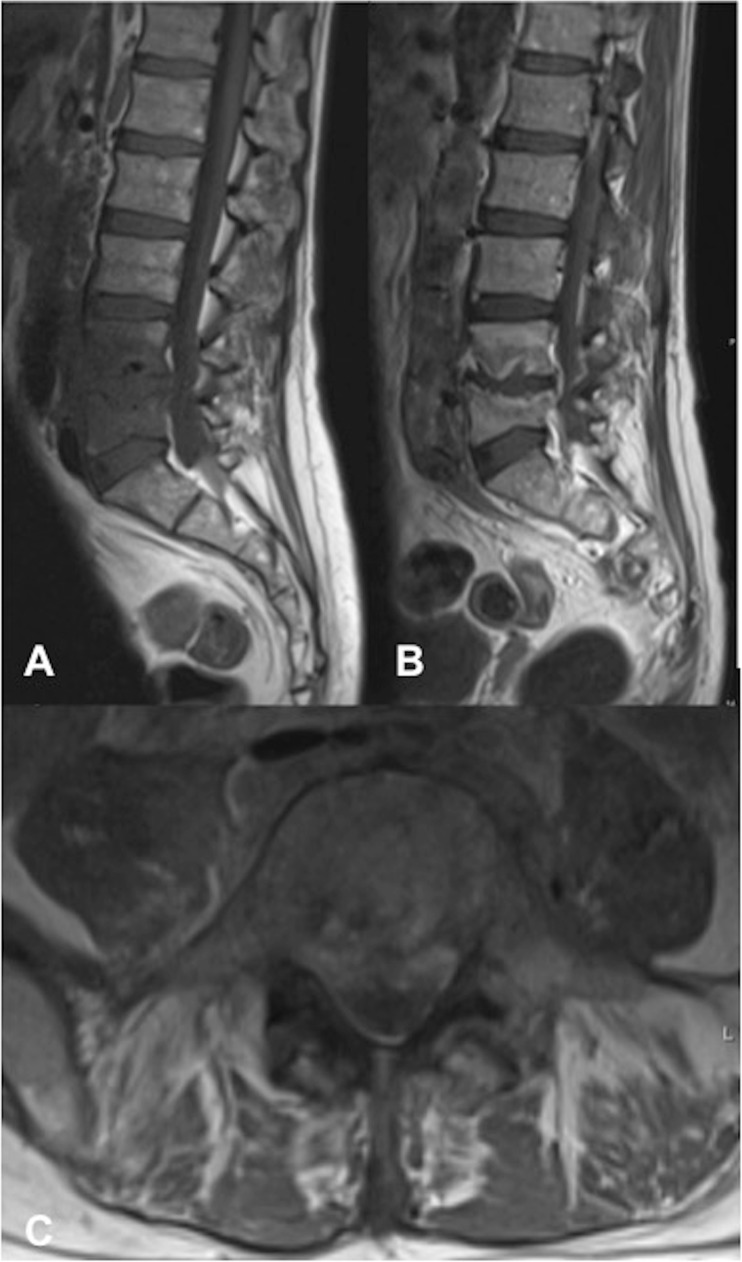
A 58-year-old man (patient 5). **a** Sagittal T1, **b** T1 post-contrast and **c** axial T1 post-contrast 3 months following EVAR. There is an L4/5 disc abscess with a paravertebral and epidural enhancing phlegmon extending into the lateral recesses bilaterally

Eight out of nine patients had paravertebral abscesses or phlegmon of varying sizes, which were either contiguous with or immediately adjacent to the native aneurysmal aortic sac. Five of the eight patients with paravertebral abscess formation also showed evidence of psoas abscess involvement. Only 3 out of 9 patients had an epidural component, of whom 2 had very minor non-compressive epidural phlegmon. The third patient with epidural disease had spondylodiscitis centred at L4/5 with an epidural phlegmon impinging on the traversing L5 nerve roots and exiting L4 nerve roots bilaterally in the lateral recesses and neural exit foramina respectively (Fig. 4).

### Treatment, progress and outcome

All patients received antibiotic treatment according to local microbiological advice. Four out of nine patients died within 7 months of first imaging identification of spondylodiscitis (3 patients secondary to vascular complications; 1 patient with advanced recurrent head and neck malignancy), of which 2 underwent stent graft removal and axillobifemoral bypass graft.

Of the 5 out of 9 patients who survived, only one underwent removal of the stent graft, with aortic reconstruction, vertebral debridement, decompression and L4/L5 spinal fusion. The remaining 4 patients were successfully treated conservatively ± attempted percutaneous drainage.

**Fig. 5:**
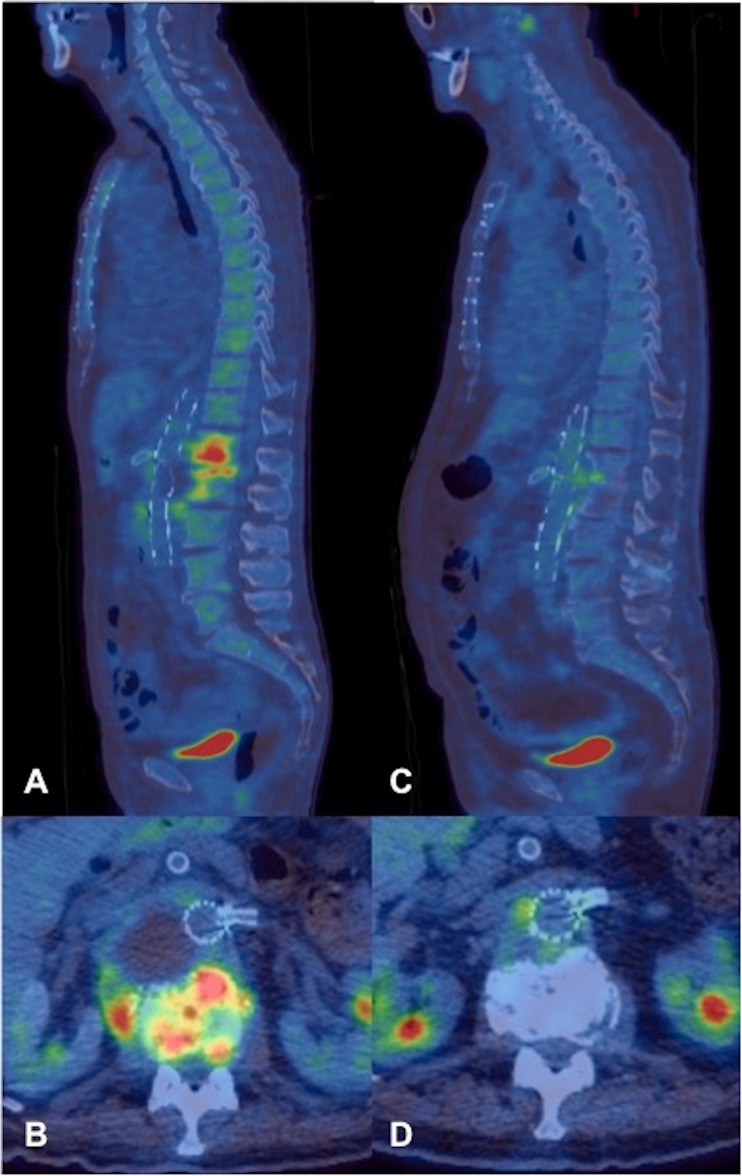
A 82-year-old man (patient 9). Serial PET/CT studies. **a** Sagittal and **b** axial PET/CT study images performed 2 months following EVAR demonstrating intense FDG uptake at level of the L1/2 disc, extending into the adjacent vertebral bodies. Note the intense anterior vertebral body uptake at L2 with further uptake extending into the paravertebral soft tissues, continuous with the dilated native aneurysm sac. **c** Sagittal and **d** axial images of the PET/CT study performed at a 12-month interval at similar levels following conservative management, demonstrating significantly reduced vertebral and paravertebral uptake. The previously demonstrated paravertebral abscess component contiguous with the native aneurysm sac had also resolved

^18^F-FDG PET/CT was adopted as the follow-up imaging modality in 3 patients owing to extensive susceptibility artefact on MRI. Two of these patients demonstrated either reduction or resolution of increased uptake at the involved disc and adjacent paravertebral disease, one of whom was treated conservatively (Fig. 5), the other undergoing debridement, decompression and L4/5 fusion (patient 5). The third patient did not attend the follow-up imaging.

**Fig. 6:**
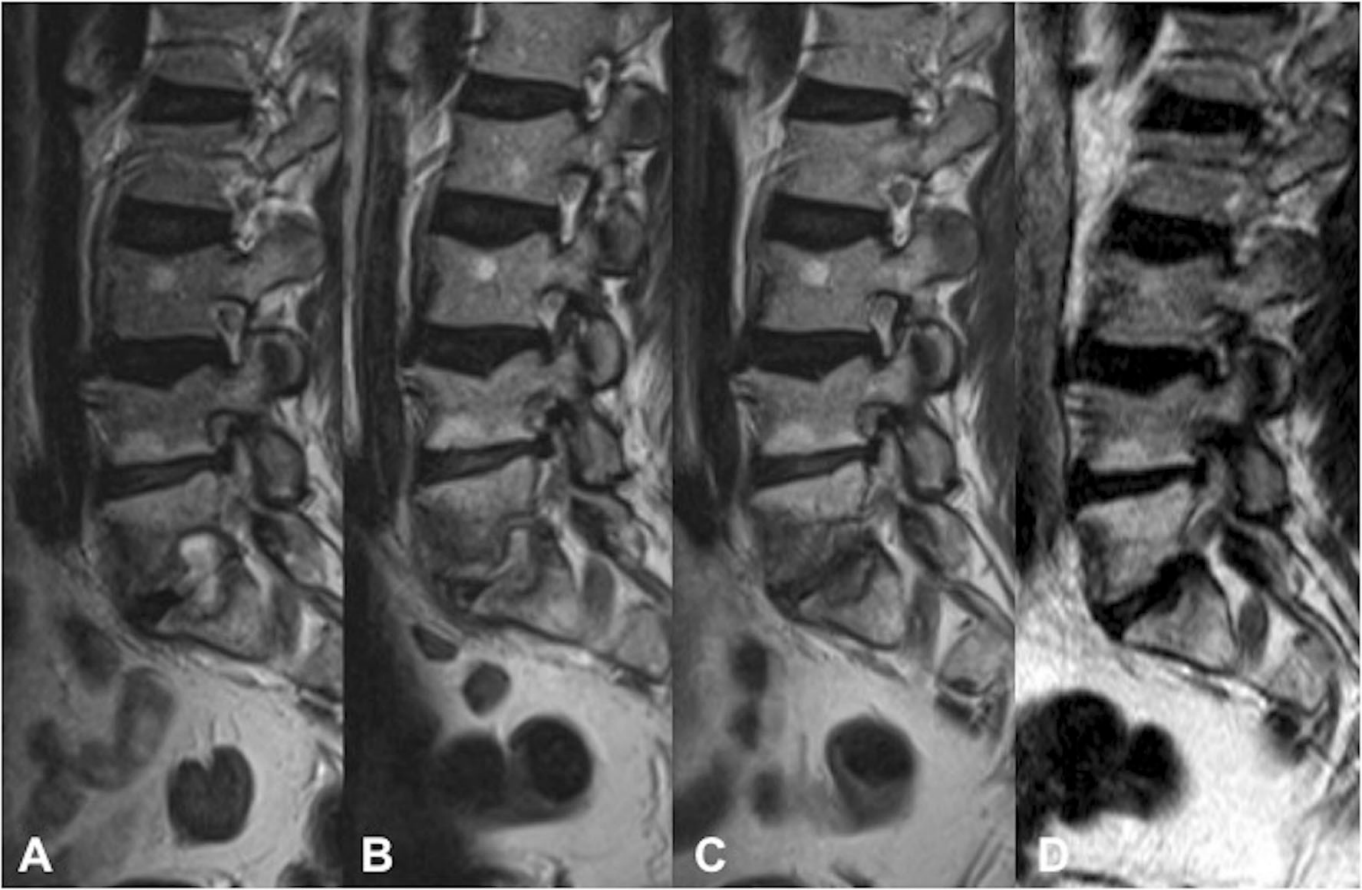
A 59-year-old man (patient 4). Sagittal T2 images from serial MRI studies demonstrating gradual resolution of an L5/S1 disc abscess extending into the adjacent endplates. **a** 3 months following EVAR, **b** 6-week interval following the initial MRI, **c** 3-month interval and **d** 14-month interval

One patient was followed up with serial MRI studies showing the resolution of disc abscess and epidural phlegmon (patient 4, Fig. 6). The final surviving patient did not undergo any interval MRI or ^18^F-FDG PET/CT following initial diagnosis of spondylodiscitis (patient 6).

## Discussion

Infection of the stent graft following EVAR is a potentially devastating but uncommon complication, with an incidence of between 0.5 and 1% and a mortality rate of 11% and 30% at 1 and 12 months in treated patients [4–8]. Although these studies have shown the tendency of infected stent grafts to result in the spread of infection to the native surrounding aortic sac and para-aortic soft tissues, it is perhaps surprising that only four previous case reports have described spondylodiscitis as a complication of EVAR given the close proximity of the vertebral column and aorta [9–12]. In all of these studies, CT imaging demonstrated features of an infected stent graft and native aneurysm sac with abscess formation contiguous with a single adjacent lumbar level with features of spondylodiscitis [9–12].

Several studies have suggested predisposing risk factors that may contribute to infection of the stent graft following EVAR, including multiple endovascular/surgical procedures, an immunocompromised state and nosocomial blood stream septicaemia [13–15]. Although specific predisposing risk factors for spondylodiscitis following EVAR are difficult to identify given our relatively small cohort, we found that all 9 patients had single-level spondylodiscitis at lumbosacral levels adjacent to the stented aorto-iliac vessels. Furthermore, 8 of the 9 patients had spondylodiscitis with adjacent paravertebral collections that were either contiguous with or immediately adjacent to the native aneurysm, 7 of whom had not only endplate, but also anterior vertebral body erosion (Fig. 1). Although the small cohort size precludes any definitive conclusions, the pattern of extensive predominantly anterior vertebral bony and paravertebral disease suggests direct spread from the adjacent infected aortic stent graft and native aneurysm sac as a likely route of vertebral column infection in most patients in our cohort. However, haematogenous spread of infection could also present with extensive anterior paravertebral disease. In only 1 patient (patient 4) was the paravertebral component not associated with the native aneurysm sac, suggesting haematogenous seeding of infection to the involved disc rather than direct spread in this particular case (Fig. 6). These findings highlight the point that patients with infected stent grafts and extensive para-aortic infection are potentially at a higher risk of adjacent level spondylodiscitis and anterior vertebral body osteomyelitis. This may also account for why relatively few patients in our cohort (3 out of 9 patients) had disease extension posterior to the epidural space (Fig. 4).

**Fig. 7:**
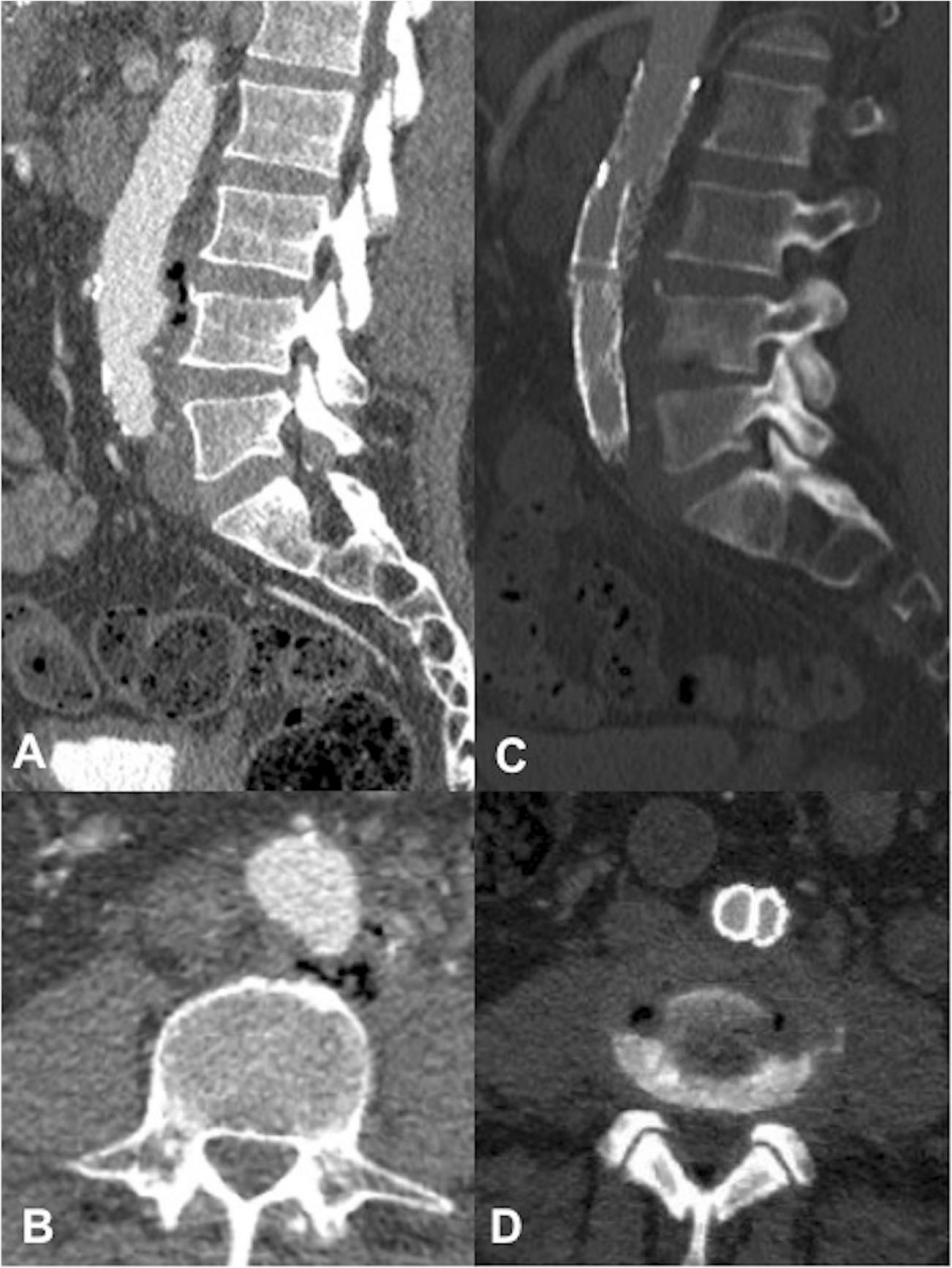
A 58-year-old man (patient 5). **a** Sagittal and **b** axial CT angiogram pre-EVAR. There is saccular aneurysmal dilatation at the L3/4 level with adjacent gas locules typical of an infective aneurysm. *E. coli* bacteraemia confirmed the infective aneurysm. **c** Sagittal and **d** axial images from routine surveillance CT 2 months post-EVAR demonstrate new L4/5 endplate erosion, anterior L4 vertebral body erosion, gas locules in the disc space and paravertebral soft-tissue density extending to the stent graft

Only one patient in our cohort had positive pre-EVAR blood culture in addition to typical saccular aneurysmal morphology, consistent with infective aneurysm (Fig. 7). The organisms identified on pre-EVAR blood cultures and post-EVAR paravertebral collection biopsies were similar (*E. coli*). Presence of an infective aneurysm and pre-existing septicaemia before any vascular intervention has been shown to be associated with the development of spondylodiscitis [16–20].

It is notable that although all 9 patients in our study demonstrated imaging features of spondylodiscitis, a positive culture was isolated in only 6 patients from a combination of blood cultures and/or CT-guided paravertebral/psoas collection biopsies. The 3 patients with no causative organism identified comprised 2 patients with culture-negative spinal biopsies and 1 patient with a culture-negative biopsy of the paravertebral collection. This is in line with previous studies that have highlighted the limited positive yield of CT-guided spinal biopsies (42–57%), and a moderately higher yield obtained from the biopsy of a paravertebral phlegmon/abscess (68%) [21–23].

With regard to the imaging assessment of spondylodiscitis following EVAR, our retrospective cohort of patients underwent varying combinations of CT, MRI and/or ^18^F-FDG PET/CT, highlighting the dilemma faced when imaging this challenging patient group. Across these modalities, the typical features of a bacterial spondylodiscitis were identified in all 9 patients—namely single-level loss of disc height, erosion/destruction of adjacent vertebral endplate cortices, signal abnormality and enhancement/tracer uptake centred on the disc involved and adjacent endplates ± paravertebral/epidural enhancing phlegmon/abscess [24]. As mentioned previously, the disproportionate extent of anterior paravertebral disease in most patients and associated anterior vertebral body erosion are suggestive of the direct spread of infection to the spine.

**Fig. 8:**
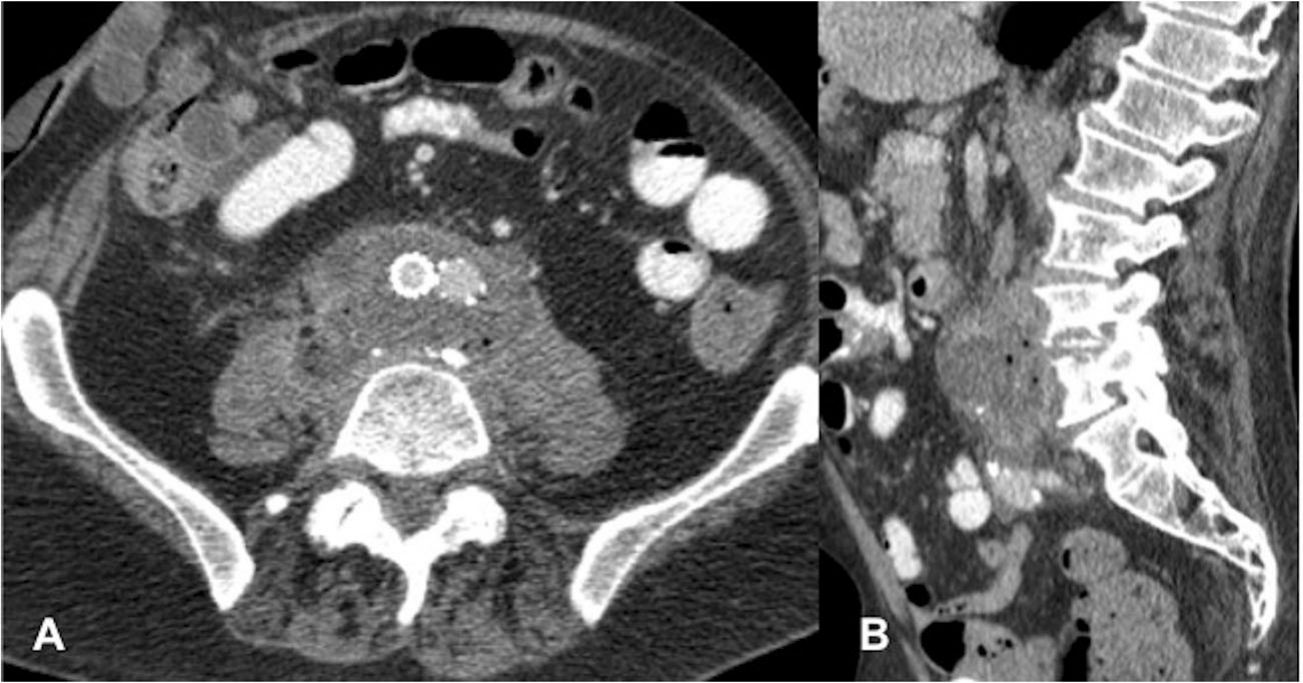
A 70-year-old man (patient 3). **a** Axial and **b** sagittal CT images 2 months post-EVAR. Note gas locules within the peripherally enhancing native aneurysm sac extending posteriorly to the vertebral column. There is a gas-containing right psoas/paravertebral abscess contiguous with the native aneurysm sac

In most cases, contrast-enhanced CT was the first cross-sectional modality performed owing to the non-specific presenting features of back/abdominal pain, fever, nausea and vomiting. More concerning was the detection of spondylodiscitis on routine post-EVAR surveillance CT in 2 patients who were asymptomatic, highlighting the often insidious course of spondylodiscitis. In 5 out of 9 patients, spondylodiscitis features were first identified on CT, demonstrating established endplate destruction ± anterior vertebral erosion, loss of disc height and paravertebral soft tissue (Figs. 1, 7). CT was also able to depict morphological changes in the native aneurysm sac compared with preoperative studies, in addition to new aortic rim enhancement and internal gas locules, indicating infection of the stent graft and aneurysm sac (Fig. 8). However, assessment of epidural disease and differentiating between paravertebral phlegmon and abscess was challenging on CT.

**Fig. 9:**
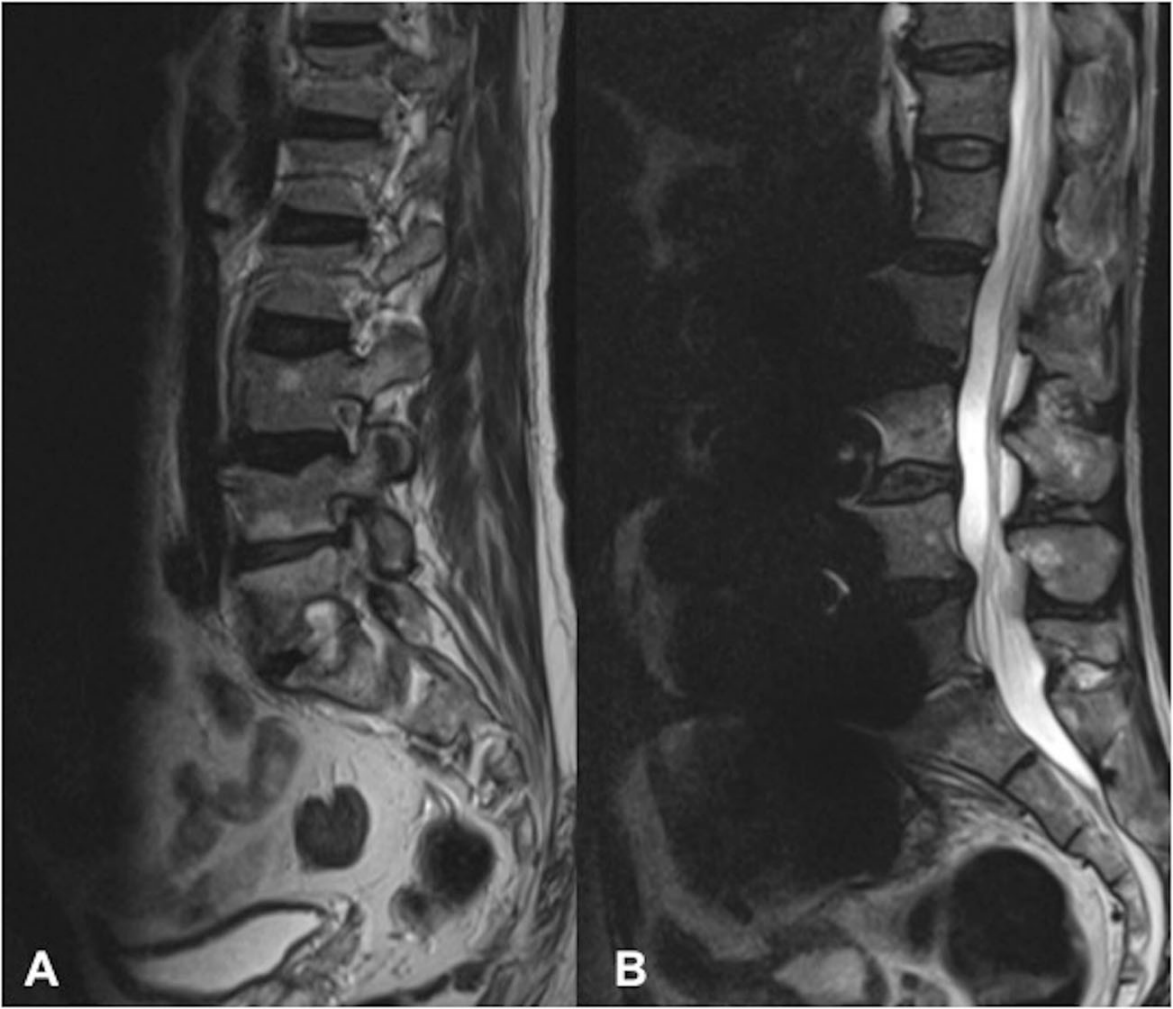
**a** Sagittal turbo spin echo (TSE) T2 in patient 4 (a 59-year-old man) with a 316L stainless steel Advanta V12 (Atrium Medical Corporation) stent in situ. Note the minimal susceptibility artefact compared with **b** sagittal TSE T2 in patient 8 (an 84-year-old man) with a 304L stainless steel Zenith Spiral (Cook Medical) stent in situ. Both sequences were obtained at 1.5 T with similar imaging parameters, differing only in the number of signal averages (3 in **a**, 2 in **b**)

Gadolinium-enhanced MRI is considered the gold standard in the assessment of spondylodiscitis, owing to its superior soft-tissue contrast permitting accurate assessment of paravertebral and epidural extension. The contrast enhancement is also superior in differentiating between a phlegmon and an abscess, in addition to the extent of marrow infiltration [24]. However, in the context of the post-EVAR patient, the added complication of susceptibility artefact from the adjacent metallic aorto-iliac stents must be considered. This may result in varying degrees of in-plane distortion (signal loss and pile-up), through-plane artefact resulting in geometric distortion, and ineffective fat suppression [25, 26]. In our cohort, the extent of susceptibility artefact was widely variable, ranging from minimal image distortion to non-diagnostic images, which precluded accurate assessment of the spine in 3 out of 9 patients. This is in large part due to the specific stent type deployed, given that currently used materials in construction of the stent skeleton may comprise nitinol (nickel titanium alloy), cobalt chromium or stainless steel alloys [12]. Nitinol-based stent grafts have the lowest magnetic susceptibility and generate minimal artefact, whereas the greatest degree of artefact is observed with stainless steel grafts, particularly those of 304L stainless steel alloys, owing to their ferromagnetic properties [27, 28]. However, the extent of artefact may be amplified or minimised significantly depending on the type of sequence and specific sequence parameters utilised. Basic methods to reduce such artefacts include the use of lower field strength, the use of spin-echo/turbo spin-echo sequences with long echo trains rather than gradient echo, and the use of short inversion time recovery (STIR) sequences for fat suppression rather than conventional spectral fat suppression [25, 26, 29]. These conventional sequences can be further optimised to reduce metallic artefacts by increasing receiver bandwidth, using a larger matrix to increase in-plane resolution and thinner slices to increase through-plane resolution (albeit at a cost of reduced overall signal-to-noise ratio (SNR)—this may be boosted with increased signal averages) [25, 26, 29]. Figure 6 shows how the application of some of these methods of optimisation allowed serial MRIs to be performed in patient 4 in the context of a 316L stainless steel stent (Advanta V12; Atrium Medical Corporation) with minimal stent-related susceptibility artefact, clearly depicting the interval resolution of spondylodiscitis. Of note, this was the only patient in whom MRI was adopted as the follow-up imaging modality and accurately demonstrated interval disease reduction. Figure 9 shows the vast difference in the extent of susceptibility artefact when compared with patient 8 in the context of a 304L stainless steel stent (Zenith Spiral; Cook Medical) with similar imaging parameters.

More recently, various three-dimensional multispectral imaging (3D-MSI) sequences have been developed, including multi-acquisition with variable-resonance image combination (MAVRIC) and slice encoding for metal artefact correction (SEMAC), which, in the context of spinal MRI in patients with metallic spinal fixation, have been shown to overcome the susceptibility of stainless steel or cobalt chromium alloys with an up to eight-fold reduction in the scan volume affected by artefact compared with conventional sequence optimisation for metal artefact reduction [25, 30–32]. The 3D-MSI sequences were not used in our patient cohort, mainly because of their retrospective nature. However, their potential in minimising susceptibility artefact and improving image quality in the context of post-EVAR spinal imaging should be explored.

In 2 patients with significant artefact on MRI precluding accurate assessment, ^18^F-FDG PET/CT proved to be of significant diagnostic value, as it accurately depicted markedly increased uptake at the spinal level involved in addition to the surrounding paravertebral disease, which could not be visualised on MRI (Fig. 3). Furthermore, ^18^F-FDG PET/CT was selected as the follow-up imaging modality in 3 patients in whom MRI had been markedly degraded by metallic susceptibility artefact from the stent graft in 2 patients, and a third patient who had undergone spinal debridement of the vertebral column involved and spinal fixation, precluding subsequent accurate MRI assessment of residual spondylodiscitis. The 2 patients who underwent follow-up ^18^F-FDG PET/CT demonstrated either reduction or resolution of spinal/paravertebral tracer uptake and a reduction in size of the paravertebral collection on interval studies (Fig. 5). These examples highlight the potential role of ^18^F-FDG PET/CT, not just for diagnosis, but also as an effective means of monitoring the progress of spinal infection in the context of previous EVAR, or indeed metallic spinal instrumentation that may preclude accurate assessment by MRI. This is supported by several studies that have shown ^18^F-FDG PET/CT to be at least equivalent to MRI in diagnosing spondylodiscitis and may be used as a means of monitoring the response to treatment [33–37]. In the absence of PET/CT facilities, other nuclear medicine modalities including ^99m^Tc-MDP bone scan and Gallium scan (particularly when combined with single photon emission computed tomography (SPECT)/CT) have also been shown to be viable alternatives for the detection of spondylodiscitis [38, 39].

Our study had a few limitations: the described imaging trends may not be definitive owing to the small sample size. The retrospective nature was another limitation, resulting in differing MR scanning protocols and imaging parameters with variable use of metal artefact reduction sequences.

## Conclusion

To the best of our knowledge, we have reported the first case series of spondylodiscitis post-EVAR—a rare complication typically occurring in the context of an infected stent graft with extensive para-aortic disease. Clinicians and radiologists alike should be alert to the possibility of spondylodiscitis as a complication of EVAR given the often non-specific and insidious clinical presentation. The imaging features of disproportionate anterior paravertebral disease and anterior vertebral body bony involvement suggest direct spread of infection to the adjacent vertebral column. The optimal imaging strategy for diagnosis and follow-up is challenging and should be directed by the type of stent graft deployed, as certain stainless steel alloys result in significant artefact that precludes accurate assessment by MRI. In such cases, ^18^F-FDG PET/CT is an accurate alternative that may be used for early diagnosis and monitoring the disease response to treatment. Experimenting with new MR sequences such as 3D-MSI should be considered.
